# Estimation the Maximum Tolerance Activity of Blood by a Simple Algorithm Method in Pediatric Differentiated Thyroid Cancer Patients Treated With Empirical Radioactive Iodine Dosing Based on Risk Stratification

**DOI:** 10.1002/kjm2.70056

**Published:** 2025-05-29

**Authors:** Yu‐Wen Chen, Cheng‐Hsun Chuang, Che‐Wei Wu, Feng‐Yu Chiang, Hon‐Man Chen, Kun‐Der Lin, Chih‐Hung Lin, Yung‐Chang Lai, Tzu‐Yen Huang

**Affiliations:** ^1^ Department of Nuclear Medicine, Kaohsiung Medical University Hospital, Faculty of Medicine, College of Medicine Kaohsiung Medical University Kaohsiung Taiwan; ^2^ Department of Otorhinolaryngology‐Head and Neck Surgery, International Thyroid Surgery Center, Kaohsiung Medical University Hospital Kaohsiung Medical University Kaohsiung Taiwan; ^3^ Department of Otorhinolaryngology, Kaohsiung Medical University, Gangshan Hospital Kaohsiung Medical University Kaohsiung Taiwan; ^4^ Department of Otorhinolaryngology, School of Post‐Baccalaureate Medicine and School of Medicine, College of Medicine Kaohsiung Medical University Kaohsiung Taiwan; ^5^ Department of Otolaryngology‐Head and Neck Surgery E‐Da Hospital Kaohsiung Taiwan; ^6^ School of Medicine, College of Medicine I‐Shou University Kaohsiung Taiwan; ^7^ Department of Surgery, Kaohsiung Medical University Hospital, Faculty of Medicine, College of Medicine Kaohsiung Medical University Kaohsiung Taiwan; ^8^ Division of Endocrinology and Metabolism, Department of Medicine, Kaohsiung Medical University Hospital, Faculty of Medicine, College of Medicine Kaohsiung Medical University Kaohsiung Taiwan; ^9^ Division of Pathology, Kaohsiung Medical University Hospital, Faculty of Medicine, College of Medicine Kaohsiung Medical University Kaohsiung Taiwan

**Keywords:** as high as safe administration (AHASA) principle, maximum tolerance activity (MTA), pediatric differentiated thyroid cancer (DTC), radioactive iodine (RAI) treatment, simple method algorithm

## Abstract

After total thyroidectomy for pediatric differentiated thyroid cancer (DTC), the subsequent radioactive iodine (RAI) treatment is a robust therapy, the dosing of which is a major concern. This study was designed to evaluate the “as high as safe administration” (AHASA) principle of RAI treatment in pediatric DTC patients based on the maximum tolerance activity (MTA) of blood to certify dosimetry via a simple algorithm method. Twenty pediatric DTC patients were enrolled and received RAI treatment empirical dosing based on risk stratification after total thyroidectomy. The MTA concentration in the blood was estimated by the modified Hänscheid equation. About 6 (30%) patients had tumors larger than 4 cm, 10 (50%) patients had lateral cervical lymph node metastasis, and 4 (20%) patients had recurrent/persistent thyroid cancer and received more than two RAIs. Five (25%) pediatric patients who had higher serum thyroglobulin antibodies levels at initial diagnosis exhibited aggressive clinical manifestations. Body weight‐based doses showed wide variability, and the Dutch recommended dose showed underdosing. In addition, lower body weight was associated with a significantly higher blood absorption dose (*R* ^2^ = 0.3849, *p* = 0.014). No severe adverse effects were observed in patients who received empirical RAI dosage according to the AHASA principle. The presentation of pediatric DTC can be advanced and aggressive. Empirical RAI dosing based on risk stratification is a simple, safe and effective method. In compliance with the AHASA principle, for prepubertal patients with very low body weight, it is necessary to calculate the blood MTA for RAI dose adjustment.

## 1 Introduction

Pediatric thyroid cancer is rare and mostly occurs in the second decade of life, during adolescence/pediatric (10–19 years) and young adult (early 20 years) stages, with the most common pathological type being differentiated thyroid cancer (DTC). However, a long lifespan and aggressive clinical course, such as larger tumors, a higher incidence of lymph node or lung metastasis, and a higher recurrence rate, are characteristics of pediatric thyroid cancer [1, 2]. Treatment for pediatric DTC relies primarily on surgery, and the initial extent of surgery is debatable due to both the favorable prognosis and the consideration of advanced disease manifestations. In addition, the surgical volume of the thyroid surgeon has a substantial impact on the outcome and prognosis [3, 4]. Therefore, radical surgery is recommended for pediatric DTC patients except for those with very early disease (those with a tumor size less than 1 cm). There is robust evidence of the benefit of radioactive iodine‐131 (RAI) treatment after total thyroidectomy in adult thyroid cancer patients [5, 6, 7, 8]. The treatment of pediatric thyroid cancer is primarily based on the American Thyroid Association (ATA) guidelines established in 2015 [5].

Determination of the RAI dose in pediatric patients may be administered by two possible methods: (1) empirically fixed prescribed activity of iodine‐131 (I‐131) employed at most treatment centers and (2) dosimetry‐based prescribed activity of I‐131 [9, 10, 11]. In general, an empirically fixed RAI dose is given according to the risk stratification of the pathological diagnosis. Therefore, the indicated RAI dosage for residual thyroid tissue ablation (30 mCi), adjuvant therapy (50–75 mCi), therapy (100–150 mCi), and metastatic palliative therapy (150–200 mCi) is the choice after total thyroidectomy [5]. The as high as safe administration (AHASA) principle for RAI treatment emphasizes administering the maximum safe radioactivity dose while ensuring radiation absorbed dose limits are not exceeded. As originally defined by Verburg et al. [12], this safety threshold is based on limiting the maximum radiation absorbed dose to the blood to 2 Gy. This approach is particularly relevant in pediatric patients, as minimizing radiation exposure while ensuring therapeutic effectiveness is crucial given their long life expectancy and increased vulnerability to late radiation‐induced side effects.

Since the overall survival of patients under the age of 55 is good for those with thyroid cancer [8], the adverse effects of RAI treatment are often a major consideration in the treatment of adolescent/young thyroid cancer patients. The acute phase reactions of RAI treatment include sialadenitis, nausea/vomiting, diarrhea, and transient cytopenia. Late‐phase reactions to RAI, such as xerostomia, dry eyes, pulmonary fibrosis, bone marrow suppression, and secondary malignancies, such as bladder cancer and leukemia, deserve the attention of clinicians [5, 6, 7, 8]. Therefore, in the 2020 Dutch recommendation for pediatric DTC [6], 100 MBq/kg is recommended for prepubertal subgroup adjustment.

The optimal RAI treatment and effective dosimetry for pediatric thyroid cancer patients, as well as the essential adjustment of the RAI treatment for risk in special patient subgroups, are still controversial. The red bone marrow is a critical organ for determining the radiation absorption dose after RAI treatment, and the exposure dose cannot be directly measured. The concentrations of I‐131 in blood and most organs are thought to be similar to those in red marrow [13, 14]. The absorbed dose in the blood represents a first‐order approximation of the radiation absorbed dose to the hematopoietic system, making it a better means to quantify the exposure from therapy than the total amount of activity administered. Blood dosimetry was introduced by Benua et al. [15], where the maximum tolerance activity (MTA) of the blood dose was restricted to 2 Gy (200 rad) while maintaining whole‐body retention at 4.4 GBq (120 mCi) at 48 h and pulmonary uptake of 3 GBq (80 mCi) at 24 h [15, 16]. Several factors, such as patient body mass and renal clearance, determine radiation exposure and are characteristic of heterogeneity [17]. Prior to the European Association of Nuclear Medicine (EANM) guidelines, complete blood dosimetry before RAI treatment was not practiced as part of the standard operating procedures at most medical centers [18]. Based on previous studies [19, 20] and clinical experience, this study used empirically fixed RAI doses in pediatric thyroid cancer patients and evaluated the MTA in the blood (AHASA principle) via a simple algorithm.

## 2 Methods

Twenty pediatric patients diagnosed with thyroid cancer were enrolled over an 8‐year period (2012 to 2020) at an academic tertiary referral hospital, Kaohsiung Medical University Hospital (KMUH), Taiwan. All patients received total thyroidectomy, including five patients who underwent surgery in the hospital in the adjacent area and were referred to KMUH for lymph node dissection and postoperative RAI treatment. The exclusion criteria included patients who did not complete treatment and who failed to complete follow‐up (*n* = 0). At our institution, cervical ultrasonography with/without cytology and cervical computed tomography (CT) with/without contrast agent were routinely performed before thyroid surgery.

The dosage of RAI was empirically determined by a multidisciplinary team consisting of endocrinologists, head and neck surgeons, endocrine surgeons, pathologists, and nuclear medicine specialists. The team evaluated the patients' risk levels primarily based on the ATA pediatric thyroid cancer guidelines, considering multiple factors including tumor size, histological subtype, aggressive features (e.g., vascular invasion, extrathyroidal extension), presence and extent of cervical lymph node metastasis (central and/or lateral compartment involvement), serum levels of thyroglobulin (Tg) and thyroglobulin antibodies (TgAb). After a comprehensive assessment of these clinical and pathological parameters, each patient was categorized into a risk group, and an empirical RAI dose (50, 100, 150, or 200 mCi) was selected accordingly. For certain subgroups—particularly younger patients, those with lower body weight (indicative of lower blood volume), and those with compromised renal function—further dose adjustments were individually considered by the multidisciplinary team based on clinical judgment and safety considerations. Due to the inherent complexity and patient‐specific variations in clinical presentations, a rigid dosing algorithm was intentionally avoided to allow flexibility and personalized care.

The radioiodine dosage is suggested to be 100 MBq/kg in the prepubertal population. From 4 to 6 weeks after surgery, patients were prepared for RAI administration either by thyroid hormone withdrawal or by recombinant human TSH (rhTSH) stimulation prior to oral intake of I‐131. Patients receiving rhTSH had serum rhTSH levels greater than 130 IU/mL after intramuscular injection. A low‐iodine diet was routinely recommended for patients while they awaited RAI treatment. Pregnancy test results were obtained for all women of childbearing age. In this study, blood absorbed dose per MBq was calculated exclusively for patients who underwent rhTSH preparation (*n* = 15); patients prepared via thyroid hormone withdrawal (*n* = 5) were not included in the blood dose estimation.

All patients with an RAI dose greater than 100 mCi received their administrations within the radiation protection ward of the hospital to eliminate radiation exposure to other tissues and to minimize contamination under regulation. During hospitalization, water intake up to 3000 cc per day was encouraged. Considering legal regulations, the discharge criterion was an exposure dose less than 30 μSv/h at a 1‐m distance, usually after 72 h of hospital stay. The equipment in the special ward had a remote monitoring system (RMS) for patient safety care. The postoperative follow‐up protocol included routine visits with the surgeon and endocrinologist and a multidisciplinary team decision on the appropriate dose of radioactive iodine within 1 month of surgery. RAI treatment was typically administered an average of 2.5 months after surgery, and patients were followed up with routine visits every 3–6 months. Post‐RAI imaging included neck/chest planar imaging, whole‐body imaging, and SPECT/CT (GE, 8DST) on the eighth or additional fifth day in the Department of Nuclear Medicine. Imaging with stage assistance was interpreted by an experienced nuclear medicine specialist.

Thereafter, regular follow‐up data comprising serum levels of Tg and TgAb as well as neck ultrasonography images were used to monitor treatment response or recurrence during thyroxine replacement treatment. Remission was defined as the absence of clinical evidence, absence of radiological evidence of disease, or undetectable levels of serum Tg or TgAb 6 months to 1 year after late RAI treatment. Persistent disease was defined as the absence of remission. Recurrent disease was defined as histological, cytological, radiological, or biochemical evidence of disease after remission.

The Medical International Radiation Dose (MIRD) algorithm for blood dosimetry assumes that radioiodine whole‐body activity decays exponentially and that 14% of the whole‐body residence time can be attributed to the blood. The simple method equation for determining the MTA level in blood was defined as [19]

$$\frac{D_{\text{Blood}}}{A_{0}}\left(\frac{\mathrm{mGy}}{\mathrm{MBq}}\right)=-\left(\frac{15.12}{\mathrm{BVL}\left(\mathrm{mL}\right)}+\frac{0.0188}{{\left(\mathrm{wt}\left(\mathrm{kg}\right)\right)}^{\frac{2}{3}}}\right)\times \frac{t\left(h\right)}{\ln\left(R\left(t\right)\right)}$$

where *D*_blood_ is the mean blood absorbed dose; *A*_0_ is the administered activity; BVL is the blood volume; and wt is the patient's weight. Whole‐body retention was estimated by the retention *R*(*t*) at *t*(h) hours after I‐131 administration.

This working formula is based on a compartment model and is composed of two parts: blood volume and residual time related to the slope of renal clearance. The mean of the absolute deviations between the estimates and actual blood doses was 14% if external whole‐body counting was performed on Day 1 or 2 after radioiodine administration [19]. In this retrospective study, we modified the total body residual time as the natural logarithm ratio of exposure doses at the 2‐ and 67‐h points in 1 m during a 65‐h period [20]. In the mathematical exponential model, the natural logarithm of the ratio between two‐point measured exposure radioactivity during a period of time represents the residual time. The residual time was relatively consistent under normal renal clearance conditions and with a lower tumor burden. Because complete blood dosimetry is not practiced, application of a simple algorithm method was essential after the administration of an empirically fixed dose of RAI, which was based on risk stratification.

Ethical approval for this study was obtained from Kaohsiung Medical University Hospital Institutional Review Board (KMUHIRB‐SV(I)‐20220027). Informed consent was waived due to the retrospective nature of the study, with all patient data anonymized and confidentiality strictly maintained throughout analysis and reporting.

The percentage, mean value, and standard deviation were calculated using Microsoft Excel 2016 (Microsoft Corp., Redmond, WA, USA).

## 3 Results

### 3.1 Characteristics of Pediatric Thyroid Cancer Patients

The data are summarized in Table 1; for more detailed patient‐level information, please refer to Table S1. Among the 20 pediatric patients, 13 were females, and 7 were males. The ages of the participants ranged from 11 to 20 years (mean ± SD 16.6 ± 2.3 years). Four patients were prepubertal girls/boys. Two (10%) patients had a family history of thyroid cancer. The body mass index (BMI) ranged from 15.9 to 49.6 kg/m^2^, and all patients had normal renal function. The main tumors were located in the right, left, and isthmus lobes in seven, six, and two patients, respectively. The main tumor sizes ranged from 1.0 to 5.5 cm (mean ± SD 3.2 ± 2.1 cm).

**TABLE 1:** Clinical and pathologic characteristics and follow‐up in pediatric differentiated thyroid cancer patients.

Total 20 cases	*N* (%)
Sex: Female/male	13 (65%)/7 (35%)
Age	11–20 years old (mean ± SD 16.6 ± 2.3)
Prepuberty (< 14 years old)	4 (20%)
Family history	2 (10%)
Tumor localization	
Right/left/isthmus	7 (35%)/6 (30%)/2 (10%)
Prior s/p thyroidectomy	5 (25%)
Tumor size	
< 2/2–4/> 4 cm	2 (10%)/11 (55%)/6 (30%)
Multiple foci	1 (5%)
Histopathology and subtype	
Papillary thyroid cancer	18 (90%)
Classic type/follicular variants/sclerotic variants	13/4/1
Follicular thyroid cancer	2 (10%)
Available BRAF test	10
Positive/negative	3 (30%)/7 (70%)
Not available	10
Cervical lymph nodal metastasis	
Positive LNs	14 (70%)
Central (N1a)/Lateral (N1b)	4 (20%)/10 (50%)
Negative LNs (N0)	6 (30%)
RAI preparation	
Withdrawal method/rhTSH method	5 (25%)/15 (75%)
RAI treatment	
50/100/120/150/200 mCi	1/6/2/14/1
One course/two courses of RAI	16 (80%)/4 (20%)
Final stage	
Stage I/stage II (lung metastasis found by RAI)	19/1
Long term follow‐up	
Remission/recurrence/persistent	16/3/1

About 18 (90%) patients had papillary thyroid cancer, 13 had the classic type, 4 had the follicular variant, and 1 had the sclerotic variant; 2 (10%) patients had follicular thyroid cancer. BRAF genetic analysis was available in 10 patients; 3 (30%) were positive, and 7 (70%) were negative. The postoperative lymph node metastasis status was N0, N1a, and N1b in 4, 10, and 6 patients, respectively. Indeed, aggressive growth of pediatric thyroid cancer at this institution has been demonstrated.

### 3.2 RAI Preparation, Treatment, and Outcomes

Four to 6 weeks after surgery, following the standard protocol of RAI preparation, 5 (25%) had the withdrawal methods, and 15 (75%) patients received the rhTSH method before oral RAI.

Based on the nature of the tumor and the presence of lymph node metastasis in the pathological report, the empirically fixed RAI dosage was determined by risk stratification as 50, 100, 150, or 200 mCi. Of the 24 RAI treatments, all but one young boy received 50 mCi, and the other patients all received a dose ranging from 100 to 200 mCi. Four patients (20%) received two RAI treatments, the dose of which was at least 100 mCi.

A 5 (25%) of the 20 patients, all females, had higher serum TgAb levels and undetectable Tg levels at initial diagnosis. Of the five patients, one patient received RAI for residual cervical lymph node metastasis after surgery, and two patients developed cervical lymph node recurrence and subsequently received treatment after surgery and the first RAI treatment. One patient received RAI twice (100 and 150 mCi) in 2013 and 2016, respectively, at our hospital. The other patient received a second RAI treatment at our hospital at a dose of 150 mCi within 1 year of each RAI treatment. The findings in this subgroup of pediatric thyroid cancer patients suggested that higher cervical lymph node recurrence was related to a higher serum titer of TgAb (underlying thyroiditis).

**FIGURE 1:**
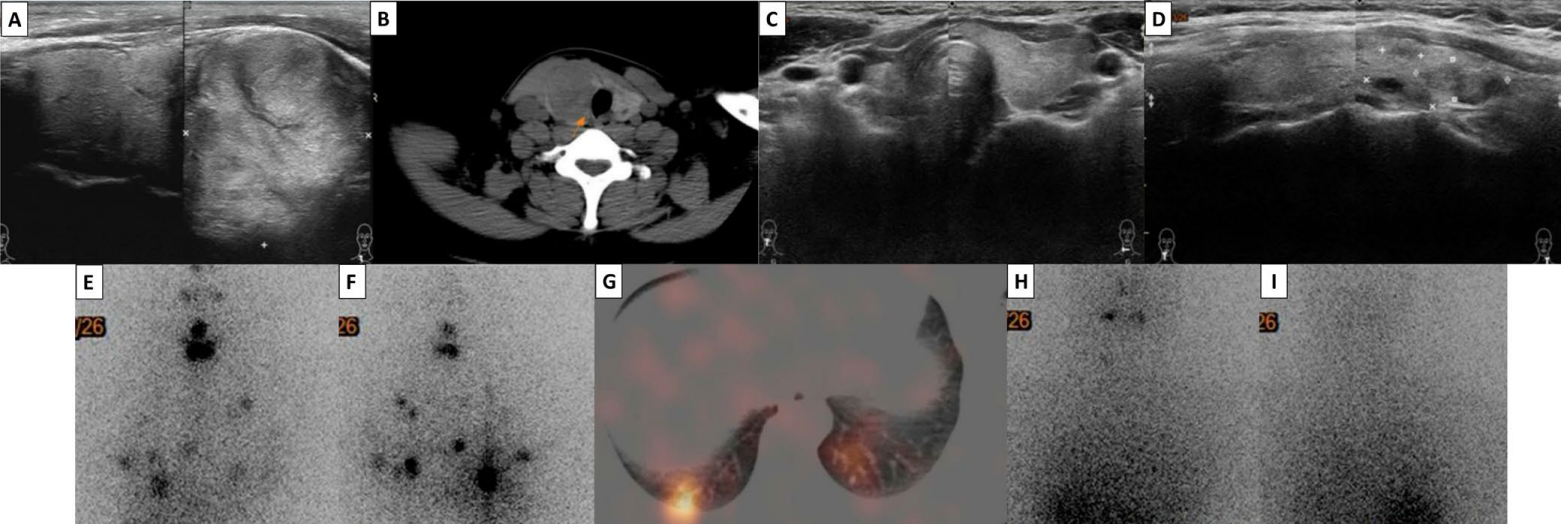
(A–C) An 18‐year‐old female with a large right thyroid nodule (4 cm) underwent right lobectomy in 2012. (D) Follicular thyroid cancer located in the left thyroid lobe was diagnosed in 2019 after several years of neck ultrasonography follow‐up. (E–G) The patient received an RAI dose of 150 mCi after complete thyroidectomy, and multiple pulmonary metastases were observed on post‐RAI imaging. (H and I) An additional RAI dose of 200 mCi was administered, and the post‐treatment scan appeared negative.

Nineteen patients had no distant metastases during the follow‐up period, and their final stage of thyroid cancer was stage I. In particular, an 18‐year‐old young female had a large right neck mass (4 cm) that was diagnosed as a benign entity after a right thyroid lobectomy in 2012. Unfortunately, follicular thyroid cancer located in the left thyroid lobe was diagnosed in 2019 after several years of neck ultrasonography follow‐up. The patient received an RAI dose of 150 mCi after complete thyroidectomy, and multiple pulmonary metastases were observed post‐RAI imaging. An additional RAI dose of 200 mCi was administered due to the elevated serum Tg titer in the next year, and the tumor showed remission (Figure 1).

**FIGURE 2:**
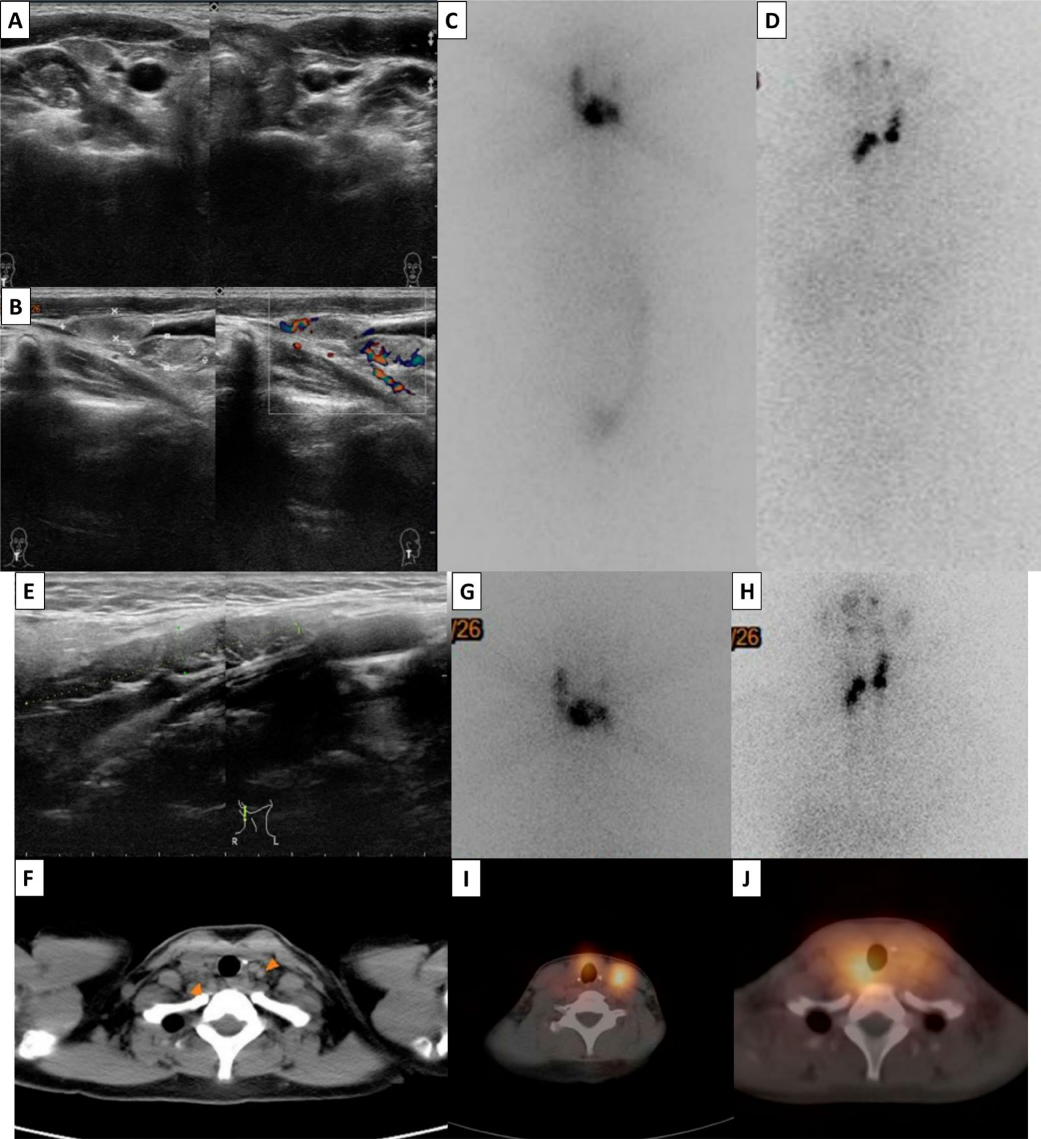
A case with persistent disease. An 11‐year‐old prepubertal girl with papillary thyroid cancer underwent total thyroidectomy and was transferred to our hospital. (A and B) Ultrasonography showed no lesions in the postoperative thyroid bed, but there were massive cervical lymph node metastases on both sides. (C and D) An RAI dose of 120 mCi was administered. Post‐RAI imaging showed good iodine avidity in the right cervical metastatic lymph nodes. (E and F) During the follow‐up the next year, cervical metastatic lymph node progression was noted in ultrasonography and a computed tomography scan. (G–J) After bilateral neck lymph node dissection, the patient received another RAI dose of 150 mCi. The residual left lower cervical lymph node avidity was found on post‐RAI imaging.

Three (15%) recurrent thyroid cancer patients and one (5%) persistent thyroid cancer patient had a second RAI after long‐term follow‐up. The younger prepubertal girl in this study (11 years old) underwent total thyroidectomy at another hospital to remove bilateral multifocal (more than 10 nodules) thyroid tumors and was diagnosed with papillary thyroid cancer. The patient was then transferred to our hospital, and ultrasonography revealed no lesions in the postoperative thyroid bed; however, there were massive cervical lymph node metastases on both sides. The RAI dose was adjusted from 150 to 120 mCi because of the patient's lower body weight (35.4 kg). Post‐RAI imaging revealed good iodine avidity in the right cervical metastatic lymph nodes. However, the patient had poor compliance with thyroxine replacement treatment. During the follow‐up year, cervical metastatic lymph node progression was noted, and bilateral neck lymph node dissection was performed. The patient received another RAI dose of 150 mCi after surgery, and residual left lower cervical lymph node avidity was found via post‐RAI imaging (Figure 2).

### 3.3 The Principle of the AHASA for RAI Treatment and the Estimated MTA of Blood From Pediatric DTC Patients

**FIGURE 3:**
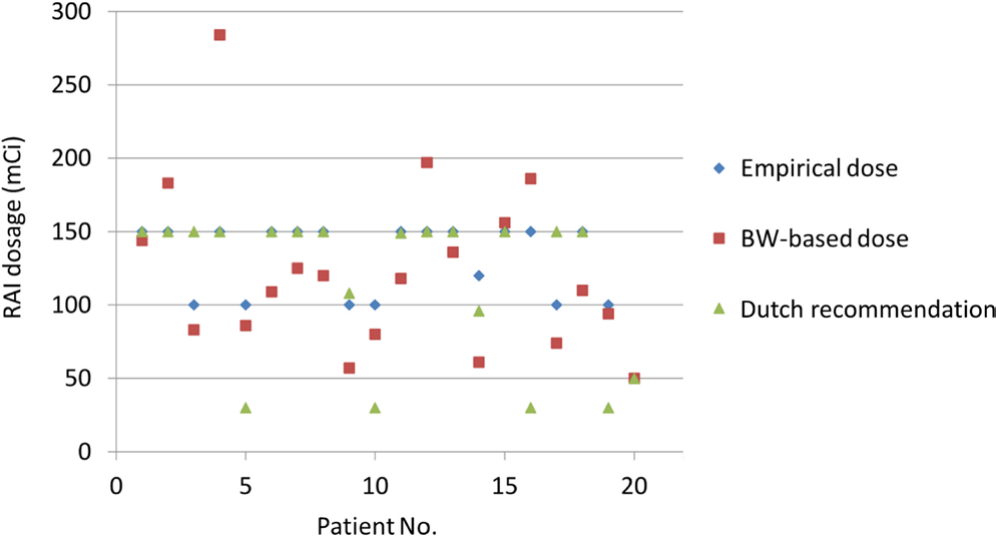
Comparison of the RAI dosages with empirical therapy (the actual dose received by patients), body weight (BW)‐based dosing, and the recent Dutch recommendation for dose adjustments. All data points represent patients prepared with rhTSH.

To present the differences in the recommended RAI doses for different dosing strategies, Figure 3 shows the RAI dosage with empirical therapy (the actual dose received by patients in this study, mean ± SD, was 131 ± 29 mCi), body weight‐based dosage (mean ± SD, was 123 ± 57 mCi), and the Dutch recommendation for pediatric DTC in 2020 for dose adjustment (mean ± SD, was 113 ± 50 mCi). The dosimetry‐based RAI dose was based on the formula body weight/70 kg × empirical dose of the adult. According to the Dutch recommendation for pediatric DTC in 2020 [6], 100 MBq/kg (T3b/N1: maximum of 5550 MBq; T4/M1: maximum of 7400 MBq) is recommended for prepubertal subgroup adjustment.

In this study, body weight‐based adjusted RAI doses exhibited wide variability (35–135 kg; 0.5 to 1.6 times). For example, for the heaviest girl (135 kg), the RAI dose was calculated to be 245 mCi (intermediate risk), but this is not clinically feasible. The dose of the Dutch recommendation was much lower than the empirical dose in four patients and even lower than the BW‐based dose, likely leading to underdosing and being contrary to the AHASA principle. No severe adverse effects were observed in patients who received empirical RAI dosage with the AHASA principle. The empirical RAI dosage based on risk stratification is a simple and safe method, and we retrospectively demonstrated an effective therapeutic response (nearly 80% achieving remission).

**FIGURE 4:**
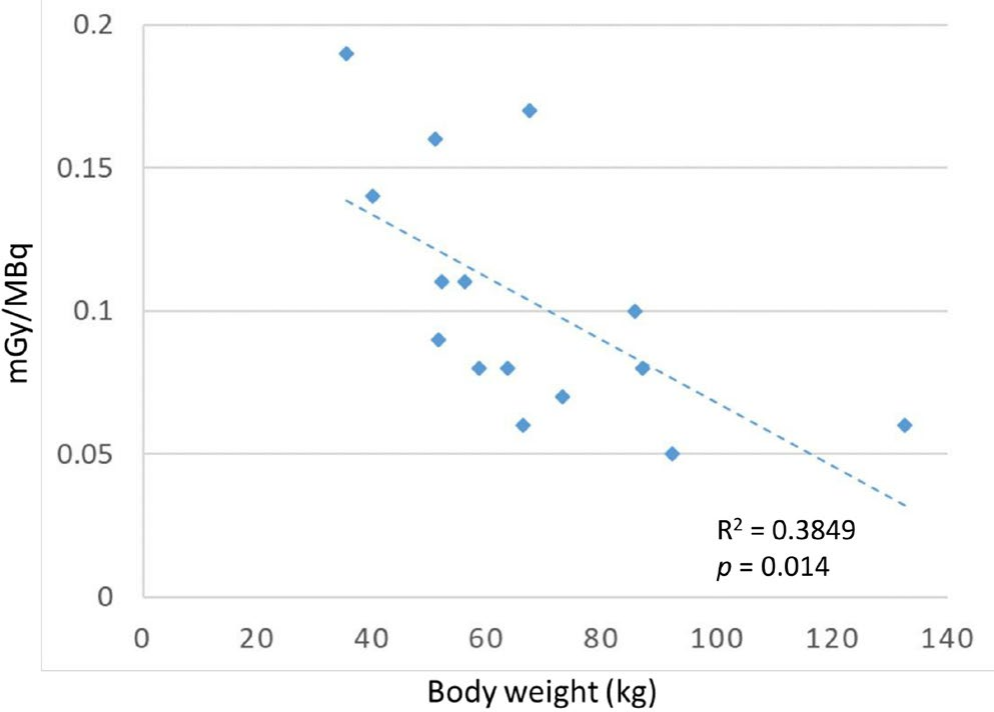
Estimation of the MTA (< 1 Gy): The relationship and the regression line between body weight (kg) and blood dosimetry (mGy/MBq). Data shown represent only the subgroup of patients who underwent rhTSH preparation. The correlation coefficient (*R* ^2^) was 0.3849, with a statistically significant *p* value (*p* = 0.014).

In RAI treatment, the MTA of the blood and bone marrow is not allowed to be over 2 Gy [5, 6]. We calculated the MTA of blood based on the modified Hänscheid equation, and the value ranged from 0.06 to 0.19 mGy/MBq, which did not exceed 1 Gy in any patient. In Figure 4, body weight and the corresponding blood dosimetry data are presented exclusively for the 15 patients prepared with the rhTSH method, and the regression line demonstrating their correlation is shown. This result indicated that lower body weight (i.e., lower blood volume) was associated with a significantly higher blood absorption dose (*R* ^2^ = 0.3849, *p* = 0.014). Therefore, for prepubertal patients who have extremely lower body weights, the MTA of blood should be calculated, the RAI dose adjusted, and the AHASA principle used as much as possible.

## 4 Discussion

Twenty pediatric patients were analyzed in this study. These results showed that the presentation of pediatric DTC is advanced and aggressive (Table 1). Five (25%) pediatric patients who had higher serum TgAb levels at initial diagnosis had aggressive clinical manifestations. According to the treatment experience of individual patients (Figures 1 and 2), the AHASA principle is applicable and recommended for the treatment of pediatric thyroid cancer. Compared to BW‐based doses, which have wide variability, using the Dutch recommended dose may be underdosing, and an empirical RAI dosage based on risk stratification is a simple, safe, and effective method (Figure 3). In addition, lower body weight was associated with a significantly higher blood absorption dose (*R* ^2^ = 0.3849, *p* = 0.014) (Figure 4). In compliance with the AHASA principle, for prepubertal patients with very low body weight, it is necessary to calculate the blood MTA for RAI dose adjustment.

In the publication by Mollen et al. [21], an analysis of 70 pediatric patients with papillary thyroid carcinoma (PTC) revealed that *BRAF* mutations accounted for 55% of the cases, while *RAS* mutations were present in 30% of cases. According to other genetic studies, *RET/PTC* translocations are more common than *BRAF* mutations [1, 8]. Our study showed that 10 patients had a *BRAF* genetic examination. Only 3 (30%) patients had positive results, which was much lower than the 60% in adults.

The pathophysiology of pediatric thyroid cancer is related to autoimmune disease, iodine deficiency, and prior radiation exposure [8]. Our hospital is an academic tertiary referral hospital; therefore, we receive many referral cases with higher severity and complexity of diseases compared to the same region. Based on informal statistics, as our location in Kaohsiung is by the seaside and without any major radiation accidents, the majority of our patient population consisted of PTC patients. However, there has not been any large‐scale thyroid cancer screening of adolescents in this region, resulting in a significantly higher severity of disease when it does occur compared to areas with screening. Five (25%) pediatric females in this study had high serum TgAb levels (419 to 7345 IU/mL) and undetectable serum Tg at initial diagnosis, and most of them had underlying Hashimoto's thyroiditis (HT). Since Dailey's first report in 1955 [22], several studies have investigated the coexistence of autoimmune thyroid disease and DTC [23]. HT and PTC share similar biomolecular features, such as RET/PTC rearrangements and the expression of p63/Akt proteins. However, it is still unclear whether HT and PTC are causally related. Retrospectively, several studies have demonstrated the prognostic significance of the histological presence of HT or the serological detection of TgAb [24, 25, 26, 27]. In this study, patients with high TgAb levels coincidentally had more cervical lymph node metastases. To examine the relationship between the incidence of thyroid cancer and the presence of thyroiditis among adolescent individuals, we propose a focus on elucidating their potential correlation in future research [28].

Applying an empirical dose of RAI after radical thyroid surgery based on the AHASA principle resulted in cancer remission in most pediatric DTC patients, showing favorable outcomes. Currently, empirical RAI dosimetry is prescribed based on two to seven 30‐mCi units, irradiating potentially residual malignancy according to risk classification [7]. The rapid clearance of nontargeted I‐131 from the body after RAI treatment is an important issue. In this study, 80% of the pediatric patients who underwent RAI treatment had an exposure dose below 10 μSv/h/m at the 65th hour after oral radioiodine, especially in patients who received rhTSH injection. The heterogeneity of the blood absorbed dose results from differences in patient size and renal function. Previous studies have shown that one‐third of elderly patients (more than 70 years old) with hypothyroidism have a blood MTA greater than 2 Gy with a RAI dose of 200 mCi [29]. Traditional measurement methods require whole‐body and blood measurements taken over 4 days to estimate the complete blood dosimetry, which is laborious and difficult. With the external measurement of whole‐body retention, based on the modified Hänscheid equation [19], the estimated MTA of blood can be calculated [20]. The validated model ratios ranged from 18.0% to 53.3% between different fixed RAI dosages (100, 150, and 200 mCi) and residual exposure doses (10, 20, 30, 40, and 50 μSv/M/h). As with the previous results, the total body retention time was nearly 14% of the whole body dose at 48 h. In the Hänscheid study, the estimated blood dosimetry to actual blood dosimetry ratio was approximately 0.67, and the estimated blood dosimetry should be conservatively less than 1.3 Gy considering the actual blood dosimetry not exceeding 2 Gy [19]. In Figure 4, the estimated blood dosimetry ranged from 0.06 to 0.19 mGy/MBq among 15 pediatric thyroid patients. The MTA of blood was 1.05 Gy in the youngest prepubertal girl with persistent disease.

As fully shown in Figure 3, traditional weight‐based pediatric dose adjustment results in wide variability in the RAI dose and is not clinically appropriate. Due to differences in radiopharmaceutical kinetics compared to traditional pediatric medications, relying solely on body weight for RAI dosage calculation may lead to significant biases [30]. Excessively high calculated doses could cause unnecessary radiation exposure, whereas overly conservative doses might lead to inadequate disease control, particularly in advanced cases. Reliance solely on body weight without integrating clinical and pathological factors may compromise outcomes. Moreover, the Dutch recommendation is likely to cause underdosing problems and is not ideal for advanced/aggressive pediatric DTC. The empirical RAI dosage based on risk stratification is a simple, safe, and effective approach. For adolescent thyroid cancer patients with lung or bone metastasis, a higher RAI dosage is an option for better disease control [31]. In our patients, no significant side effects were recorded, even in patients who received two doses. Avoiding extreme dosages may reduce side effects, but further research into specific organ absorption and side effects is needed. The core concept of ideal RAI therapy is to avoid undertreatment and potential side effects caused by excessively high doses according to the AHASA principles. Due to the stunning effect of I‐131 and the difficulty in obtaining I‐123 in Taiwan, back‐calculating the MTA is necessary when pretreatment imaging cannot be performed. Using empirical doses and the modified Hänscheid equation to calculate the MTA of blood is a favorable method for determining pediatric RAI doses. However, although the AHASA principle offers practical and safe guidelines for clinical implementation, simplified models inherently carry risks of underdosing or overdosing. Fixed empirical dosages might not fully account for individual variability in body weight, tumor burden, renal clearance, and iodine kinetics, potentially leading to insufficient treatment efficacy or increased side effects. Thus, clinicians must remain vigilant and consider individualized adjustments, especially in patients with atypical clinical features or comorbid conditions. Future studies with more sophisticated, individualized dosimetry methods would be beneficial to refine dosing accuracy and further optimize therapeutic outcomes. In the future, with the help of big data and artificial intelligence, it may be possible to establish a more effective method for determining optimal RAI dosing.

Several limitations should be mentioned. First, the small number of patients enrolled in this study made it difficult to categorize and compare treatment effects, and precluded meaningful multivariate analyses. Future studies with larger sample sizes, multi‐center data, or meta‐analytical approaches are needed to further validate the robustness and generalizability of our proposed simple MTA calculation algorithm. Second, none of the patients were younger than 11 years old or lighter than 35 kg, thus limiting direct assessment of the applicability or efficacy of our algorithm in very young pediatric patients. In addition, due to the retrospective and empirical nature of this study, validation of the modified Hänscheid equation by comparison with other established dosimetry methods was not performed. Future prospective studies including younger pediatric populations and direct comparative validations against other dosimetry methods would be valuable for confirming the clinical reliability of our algorithm. Third, blood absorbed dose estimation in this study was performed solely in the rhTSH‐prepared subgroup, and no direct comparison with the withdrawal subgroup was feasible due to subgroup size constraints. Fourth, RAI dose in this study was not entirely based on risk status. Given the current lack of clear consensus on RAI dosing in adolescents with DTC, dose decisions may vary widely between institutions. However, this study provides the measurement of MTA after fixed RAI dose, thus minimizing variability related to risk stratification and guideline updates. Fifth, this was not a full dosimetry study. The MTA calculation method was used, but verification of its accuracy is beyond the scope of this study. Finally, the efficacy or optimal dose of rhTSH in pediatric patients has not been determined. Fifteen (75%) patients received a dose of half or less of the rhTSH before RAI treatment in this study. Using rhTSH may reduce MTA in the blood [32, 33], and the effect on the efficacy of RAI treatment is inconclusive.

In conclusion, the presentation of pediatric DTC can be advanced and aggressive. According to the treatment experience of individual patients, the AHASA principle is applicable in pediatric thyroid cancer patients. The determination of empirical RAI dosage based on risk stratification is a simple, safe, and effective method. In compliance with the AHASA principle, for prepubertal patients with very low body weight, it is necessary to calculate the blood MTA for RAI dose adjustment.

## Conflicts of Interest

The authors declare no conflicts of interest.

## Supporting information


Table S1.

## Data Availability

The data that support the findings of this study are available on request from the corresponding author. The data are not publicly available due to privacy or ethical restrictions.
