# Supplementary material for: Estimation the Maximum Tolerance Activity of Blood by a Simple Algorithm Method in Pediatric Differentiated Thyroid Cancer Patients Treated With Empirical Radioactive Iodine Dosing Based on Risk Stratification
Source: Kaohsiung J Med Sci. 2025 May 29;41(10):e70056. doi: 10.1002/kjm2.70056 (PMC12520466; doi:10.1002/kjm2.70056)
Supplement: Supplementary file 1 — Table S1. [file KJM2-41-e70056-s001.docx]

| **Supplemental Table 1. Detailed clinical characteristics, treatment parameters, and dosimetry for each pediatric differentiated thyroid cancer patient (cases 1–20)** | | | | | | | | | | | | | | | | | |
| --- | --- | --- | --- | --- | --- | --- | --- | --- | --- | --- | --- | --- | --- | --- | --- | --- | --- |
| Case # | Sex | Age | Family History | Main Tumor localization | Tumor size | Pathology | Subtype (Variant) | BRAF test | N stage | RAI  preparation | RAI Course | RAI Dose (mCi) | Final Stage | Long term follow-up | BW (kg) | MTA (mGy/MBq) | AHASA Activity (mCi, Estimated) |
| 1 | F | 17 | No | Right | >4cm | Papillary | Sclerotic | Negative | N1b | rhTSH | 1 | 150 | I | Remission | 67 | 0.17 | 318 |
| 2 | F | 18 | No | Prior surgery | >4cm | Follicular |  | N/A | N0 | rhTSH | 2 | 150/200 | II | Recurrence | 86 | 0.08 | 676 |
| 3 | F | 16 | Yes | Left | 2-4 cm | Papillary | Classic | Negative | N1b | rhTSH | 2 | 100/150 | I | Recurrence | 58 | 0.08 | 676 |
| 4 | F | 18 | No | Left | >4cm | Papillary | Classic | Positive | N1a | rhTSH | 1 | 150 | I | Remission | 133 | 0.06 | 901 |
| 5 | F | 20^#^ | No | Right | 2-4 cm | Papillary | Follicular | N/A | N0 | withdrawal | 1 | 100 | I | Remission | 60 | N/A | N/A |
| 6 | F | 17 | No | Left | 2-4 cm | Papillary | Classic | N/A | N1b | withdrawal | 1 | 150 | I | Remission | 51 | N/A | N/A |
| 7 | M | 17 | No | Right | 2-4 cm | Papillary | Classic | N/A | N1b | withdrawal | 1 | 150 | I | Remission | 59 | N/A | N/A |
| 8 | F | 17 | No | Left | 2-4 cm | Papillary | Classic | N/A | N1b | rhTSH | 1 | 150 | I | Remission | 51 | 0.16 | 338 |
| 9 | F | 13* | Yes | Isthmus | 2-4 cm | Papillary | Classic | N/A | N1a | rhTSH | 1 | 100 | I | Remission | 40 | 0.14 | 386 |
| 10 | M | 17 | No | Prior surgery | >4cm | Papillary | Follicular | N/A | N0 | rhTSH | 1 | 100 | I | Remission | 56 | 0.11 | 491 |
| 11 | F | 12* | No | Left | >4cm | Papillary | Follicular | Negative | N1b | rhTSH | 1 | 150 | I | Remission | 52 | 0.09 | 601 |
| 12 | F | 11* | No | Prior surgery | >4cm | Papillary | Classic | Negative | N1b | rhTSH | 2 | 120/150 | I | Persistent | 92 | 0.05 | 1081 |
| 13 | F | 18 | No | Right | 2-4 cm | Papillary | Classic | Negative | N1b | rhTSH | 1 | 150 | I | Remission | 63 | 0.08 | 676 |
| 14 | M | 19 | No | Prior surgery | 2-4 cm | Follicular |  | N/A | N0 | withdrawal | 1 | 120 | I | Remission | 35 | N/A | N/A |
| 15 | F | 17 | No | Right | 2-4 cm | Papillary | Follicular | N/A | N0 | rhTSH | 1 | 150 | I | Remission | 73 | 0.07 | 772 |
| 16 | M | 18 | No | Left | 2-4 cm | Papillary | Classic | Negative | N1b | rhTSH | 1 | 150 | I | Remission | 85 | 0.1 | 541 |
| 17 | M | 17 | No | Right | 2-4 cm | Papillary | Classic | Positive | N1b | rhTSH | 1 | 100 | I | Remission | 35 | 0.19 | 284 |
| 18 | F | 18 | No | Right | Multifoci | Papillary | Classic | Positive | N1a | rhTSH | 1 | 150 | I | Remission | 52 | 0.11 | 491 |
| 19 | M | 18 | No | Isthmus | <2cm | Papillary | Classic | Negative | N0 | rhTSH | 1 | 100 | I | Remission | 66 | 0.06 | 901 |
| 20 | M | 13* | No | Prior surgery | <2cm | Papillary | Classic | N/A | N1a | withdrawal | 2 | 50/150 | I | Recurrence | 70 | N/A | N/A |
| N/A = Not Applicable; RAI= radioactive iodine; BW= body weight; MTA= maximum tolerance activity; AHASA= as high as safe administration;  * Prepuberty, age younger than 14 years; # Under 20 completed years of age | | | | | | | | | | | | | | | | |  |
